# Dissection of the Genetic Basis of Yield-Related Traits in the Chinese Peanut Mini-Core Collection Through Genome-Wide Association Studies

**DOI:** 10.3389/fpls.2021.637284

**Published:** 2021-05-20

**Authors:** Xiaojing Zhou, Jianbin Guo, Manish K. Pandey, Rajeev K. Varshney, Li Huang, Huaiyong Luo, Nian Liu, Weigang Chen, Yong Lei, Boshou Liao, Huifang Jiang

**Affiliations:** ^1^Key Laboratory of Biology and Genetic Improvement of Oil Crops, Ministry of Agriculture, Oil Crops Research Institute of the Chinese Academy of Agricultural Sciences, Wuhan, China; ^2^Center of Excellence in Genomics & Systems Biology, International Crops Research Institute for the Semi-Arid Tropics, Hyderabad, India

**Keywords:** yield-related traits, mini-core collection, GWAS, diagnostic marker, peanut

## Abstract

Peanut is an important legume crop worldwide. To uncover the genetic basis of yield features and assist breeding in the future, we conducted genome-wide association studies (GWAS) for six yield-related traits of the Chinese peanut mini-core collection. The seed (pod) size and weight of the population were investigated under four different environments, and these traits showed highly positive correlations in pairwise combinations. We sequenced the Chinese peanut mini-core collection using genotyping-by-sequencing approach and identified 105,814 high-quality single-nucleotide polymorphisms (SNPs). The population structure analysis showed essentially subspecies patterns in groups and obvious geographical distribution patterns in subgroups. A total of 79 significantly associated loci (*P* < 4.73 × 10^–7^) were detected for the six yield-related traits through GWAS. Of these, 31 associations were consistently detected in multiple environments, and 15 loci were commonly detected to be associated with multiple traits. Two major loci located on chromosomal pseudomolecules A06 and A02 showed pleiotropic effects on yield-related traits, explaining ∼20% phenotypic variations across environments. The two genomic regions were found 46 putative candidate genes based on gene annotation and expression profile. The diagnostic marker for the yield-related traits from non-synonymous SNP (Aradu-A06-107901527) was successfully validated, achieving a high correlation between nucleotide polymorphism and phenotypic variation. This study provided insights into the genetic basis of yield-related traits in peanut and verified one diagnostic marker to facilitate marker-assisted selection for developing high-yield peanut varieties.

## Introduction

Peanut (*Arachis hypogaea* L.) is an important oilseed crop and a key source of oil and protein. It is grown in more than 100 countries, with a world production of 45.95 m tons from an area of 28.5 m ha (FAOSTAT, 2018). It is expected that the current demand of peanut will increase at a much higher growth rate in the next decades (Foley et al., 2011; Tilman et al., 2011). This poses a big challenge to researchers in enhancing peanut productivity at a faster pace. Genomics-assisted breeding (GAB) has been successfully demonstrated to facilitate the process of improved varietal development in peanut (Pandey et al., 2020). Identification of stable major loci and closely associated markers for yield-related traits in peanut will provide applicable opportunity for GAB to accelerate the development of high-yield varieties.

Seed size, seed weight, pod size, and pod weight have direct impact on peanut yield. Some efforts have been made on the genetic basis of these yield-related traits. Based on temporary separated populations, such as F_2_, BC_2_F_2__:__3_, and F_2__:__3_, dozens of main effect quantitative trait loci (QTLs) were identified (Fonceka et al., 2012; Shirasawa et al., 2012; Huang et al., 2015; Chen W. et al., 2016). Based on recombinant inbred line (RIL) populations, some major effect QTLs were detected in multiple environments (Luo et al., 2018; Zhang et al., 2019; Zhou et al., 2019). Luo et al. (2018) identified two stable major QTLs located on chromosomal pseudomolecules A05 and A07 which simultaneously affected the traits of pod length (PL), pod width (PW), and hundred-pod weight (HPW). Zhou et al. (2019) reported stable major QTLs on B06 that were also pleiotropic for the above-mentioned three pod-related traits. Zhang et al. (2019) mapped a stable major QTL for hundred-seed weight (HSW) and seed length (SL) on A02 and a stable QTL for HSW and seed width (SW) on B06. Recently, Gangurde et al. (2020) identified the stable major QTLs for HPW and HSW on A05, A06, B05, and B06 using nested-association mapping populations. In addition, a few studies have reported associated loci for yield-related traits using the natural populations of peanut (Pandey et al., 2014; Zhao et al., 2017; Zhang et al., 2017; Wang J. et al., 2019). Zhao et al. (2017) identified a major SSR marker that was consistently detected to be associated with SL and HSW in different environments. Although some stable major QTLs for seed- and pod-related traits have been reported in different peanut germplasms, there is no information yet on diagnostic markers available in breeding.

As the important affected factors of yield, seed (pod) size and weight have been extensively studied in some crops, leading to better understanding of the genetic basis. A large number of QTLs were identified, and dozens of important genes were cloned. In rice, the genes that controlled grain-related traits, such as *GS3*, *GW2*, *BG1*, *D61*, *GW5*/*qSW5*, and *qGL3*/*qGL3.1*, simultaneously affected two or more traits of grain size or weight (Fan et al., 2006; Zuo and Li, 2014; Peng et al., 2015). In *Brassica napus*, the gene *ARF18* affected both seed weight and silique length (Liu et al., 2015), and three major QTLs simultaneously controlled seed weight and silique length (Yang et al., 2012; Li et al., 2015). Compared with the research progress in other crops, in peanut it is still far from comprehensively understanding the genetic factors controlling these traits.

Up to now, most of the QTLs for yield-related traits in peanut were found using bi-parental populations through linkage mapping. The bi-parental population involves only two parents that allow a limited number of meiosis, leading to limited recombination events in the population (Korte and Farlow, 2013). Compared with linkage mapping, association analysis employs natural populations to discover genomic regions associated with target traits in a relatively high resolution and unbiased manner in broad-based and diverse accessions. With the availability of high-throughput sequencing technology and the development of bioinformatics and statistical methods, genome-wide association study (GWAS) using large-scale single-nucleotide polymorphisms (SNPs) has become an important alternative in dissecting the genetic basis of quantitative traits. In addition, the reference genome sequences of both wild diploid progenitors *A*rachis *ipaensis* and *Arachis duranensis* (Bertioli et al., 2016; Chen X. et al., 2016; Lu et al., 2018), as well as the assembled allotetraploid cultivated peanut *A. hypogaea* (Bertioli et al., 2019; Chen et al., 2019; Zhuang et al., 2019), are important resources for sequence-based trait mapping and genetics research.

In order to better understand the genetic basis of yield-related traits in peanut, we sequenced 250 accessions of the Chinese peanut mini-core collection by genotyping-by-sequencing (GBS) method and investigated the phenotypic data for six yield-related traits in four environments. By exploiting the genomic variation from the population and performing GWAS for seed (pod) size and weight in multi-environments, the significantly associated loci were identified, and stable major associations were found. Two associated genomic regions were investigated for candidate gene discovery and making validated diagnostic markers available for breeding.

## Materials and Methods

### Phenotypic Statistics in Multiple Environments

A total of 250 accessions of the Chinese peanut mini-core collection were obtained from the China National Gene Bank, Oil Crops Research Institute, Chinese Academy of Agricultural Sciences, Wuhan, China. These materials were maintained by self-pollination for several generations. Detailed information of the accessions is listed in Supplementary Table 1.

For phenotyping, the population was planted in two consecutive years (2015 and 2016) at the experimental fields of Nanchong, Sichuan, China, and Wuhan, Hubei, China. The four experimental environments were designated as E1 (Nanchong in 2015), E2 (Wuhan in 2015), E3 (Nanchong in 2016), and E4 (Wuhan in 2016), respectively. A random block design with three replications was adopted for generating phenotyping data across four environments. Twelve plants per accession were planted in a 2.5-m-long single row, maintaining a 30-cm distance between rows. The traits of PL, PW, SL, SW, HPW, and HSW were measured after drying the harvested pods according to the described standard procedures (Jiang et al., 2006). The minimum, maximum, mean, standard deviation (SD), and coefficients of variation (CV) of phenotypic data for each trait in each environment were analyzed using the MEANS procedure of SAS (Schlotzhauer and Littell, 1997). The broad-sense heritability was calculated according to Hallauer and Miranda (1998) as *h*^2^ = σ^2^_*g*_/(σ^2^_*g*_ + σ^2^_*ge*_/*n* + σ^2^_*e*_/*nr*), where σ^2^_*g*_ was the genetic variance, σ^2^_*ge*_ was the interaction variance of the genotype with environment, σ^2^_*e*_ was the variance of residual error, *n* was the number of environments, and *r* was the number of replications. The multi-environment joint variances were obtained using the general linear model (GLM) procedure of SAS (Schlotzhauer and Littell, 1997). Correlation coefficients between traits across environments were calculated using the CORR procedure of SAS (Schlotzhauer and Littell, 1997).

### GBS Library Construction and High-Throughput Sequencing

The genomic DNA was extracted from a single plant of each accession using a modified cetyltrimethyl ammonium bromide method. For constructing GBS sequencing libraries, initially, 10 ug of genomic DNAs from each accession was incubated with *Mse*I (New England Biolabs, Ipswich, MA, United States), T4 DNA ligase (NEB), ATP, and the Y-adapter containing a barcode, followed by digestion at 37°C and heating at 65°C to inactivate the enzymes. Then, two additional enzymes, *Hae*III and NlaI (NEB), were simultaneously added into the same tube to further digest the fragments at 37°C. The digested fragments with ligations were purified using the Agencourt AMPure XP System, followed by PCR amplifications with purified samples and Phusion Master Mix (NEB) after adding a universal primer and an index primer to each sample. The PCR reactions were purified using Agencourt AMPure XP (Beckman) and electrophoresed on 2% (w/v) agarose gel, followed by fragment isolation of 340–410 bp (including indexes and adaptors) using a Gel Extraction Kit (Qiagen, Valencia, CA, United States). These fragments were then purified using the Agencourt AMPure XP System and were then diluted for sequencing on the Illumina HiSeq platform by Novogene-Beijing, China.

### SNP Calling and Genotype Imputation

To generate the genotype data for GWAS, the method was essentially according to Jia et al. (2013). Only the unique sequences mapped to unique locations in the progenitor genome sequences of peanut were retained for SNP detection using the software of Burrows-Wheeler Aligner (BWA, version: 0.7.8) and Genome Analysis Toolkit (GATK, version 2.4). We used Bayesian algorithm to detect the polymorphism SNP site on a population scale and calculated genotype likelihoods from reads for each individual at each genomic location and the allele frequencies in the sample. Only the SNPs that had biallelic candidate SNP loci, had minor allele frequency (MAF) ≥ 0.05, and contained genotype calls of over 50 accessions were left for subsequent imputation. Genotype imputation was conducted for the remaining SNPs with missing data using the function infer sporadic missing genotype data of Beagle software, version 3.1.0 (Browning and Yu, 2009). Furthermore, the imputed genotypes with probability of less than 60% were removed from the dataset, and each marker was required to be present in ≥80% of population individuals. The imputation of the genotypes of 250 peanut accessions reduced the missing genotype calls from 35.02 to 3.04%. Four accessions were selected from the association population and sequenced for nearly 10 times as much data as before. The specificity of the genotype data before and after imputation was assessed using the sequencing data of the four accessions as quality control (SupplementaryTable 2). Finally, the remaining SNPs were annotated using the package Annovar (Wang et al., 2010).

### Population Genetic Analysis and the Division of Geographical Regions

The final set of high-quality SNPs for the 250 peanut accessions was used to assess the population structure (*Q*), phylogenetic trees, principal component analysis (PCA), and relative kinship (*K*). The number of groups (*k*) was determined using Structure 2.2 (Pritchard et al., 2000). Five independent simulations were performed for values of *k* ranging from 1 to 10. For each simulation, 10,000 iterations before a burn-in length of 10,000 Markov Chain Monte Carlo replications were conducted. The optimal *k* value was determined by the posterior probability [LnP(*D*)] (Pritchard et al., 2000) and delta *k* (Evanno et al., 2005). The *Q* values were calculated as the probability that the genomic variation of each individual came from each population. The boundary value of membership probabilities was set as 0.75 by comprehensively comparing the results of the population structure, phylogenetic trees, PCA, and *K*. The accessions with membership probabilities ≥0.75 were assigned to corresponding groups, and the accessions with membership probabilities <0.75 in any given group were assigned to a mixed group. Phylogenetic trees were inferred using pairwise genetic distances matrix data of all individuals, calculated by TreeBest v1.9.2^1^. The bootstrap was set to 1,000 times to assess the branch reliability. PCA was performed using the package GCTA^2^. Tracy–Widom test was used to determine the significance level of the eigenvectors.

We divided several geographic regions in China into southern, northern, and central regions. The northern China group included the provinces of Shandong, Hebei, Henan, Liaoning, Beijing, Heilongjiang, Shanxi, and Shaanxi. The southern China group included the provinces of Guangdong, Guangxi, Fujian, Zhejiang, Guizhou, Yunnan, and Hainan. The central China group included the provinces of Hubei, Sichuan, Hunan, Anhui, Jiangxi, and Jiangsu.

### Genome-Wide Association Analysis and Candidate Gene Discovery

The software GEMMA^3^ was used to perform the GWAS analysis. Firstly, the models of the GLM (*Q*), MLM (PCA + *K*), and MLM (*Q* + *K*) were compared, and quantile (QQ) plots were used to evaluate the most suitable model for association analysis. The MLM (PCA + *K*) model exhibited the best performance to eliminate false positives and was selected for GWAS. We defined the whole-genome significance cutoff as the Bonferroni test threshold. The threshold was set as −log_10_(0.05/105814) = 6.33. Those with *P* < 4.73 × 10^–7^ were defined as significant trait-associated SNPs. The Manhattan and QQ plots were drawn using R (Turner, 2018). To identify the putative candidate genes associated with each trait, the regions of 1.3 Mb upstream or downstream of associated SNPs were used to search genes according to the linkage disequilibrium (LD) decay of the population. The genes were defined as candidate associated genes within the genomic regions as described in Wei et al. (2015). The squared correlation coefficient (*r*^2^) was used to assess the LD using the Tassel 5 software (Bradbury et al., 2007). The significance of *r*^2^ was calculated based on Fisher’s exact test. The *r*^2^ was calculated between each pair of SNPs on the same chromosome (Weir, 1996). Decay of *r*^2^ value was analyzed across all chromosomes (Alhaddad et al., 2013). The value where *r*^2^ dropped to 50% of its maximum was set as the background level (Wei et al., 2015). The decay of LD with genetic distance was estimated by interval rather than marker pairs individually to reduce the influence of outliers as per Mather et al. (2007). We combined *r*^2^ values with *P* < 0.05 of all chromosomes into an interval series of 0–0.5, 0.5–1, 1–1.5, 1.5–2, 2–2.5, 2.5–3, 3–3.5, 3.5–4, 4–4.5, and 4.5–5 Mb based on marker distance. We estimated the averaged *r*^2^ for each interval and assumed the *r*^2^ value with 0-Mb marker distance to be 1 as previously described (Yan et al., 2009). The non-linear regression function was deployed to fit the trend of LD decay.

### Validation of Variation in the Diagnostic Marker

The diagnostic SNP marker was amplified by PCR and sequenced to determine the base variation. The sequences of the primer were from the peanut reference genome of Tifrunner^4^. Primers were designed using Primer 3 software^5^ based on the flanking sequences of the SNP variant and the specificity of the primers. The primer left sequence for PCR was GAGATAAATTTCTTTCATATTTTACG, and the primer right sequence for PCR was TTGTCCCCTGATCCAGCATA. PCR product was sequenced using the primer of GAGATAAATTTCTTTCATATTTTACG.

## Results

### Phenotypic Diversity

Six yield-related traits, including PL, PW, SL, SW, HPW, and HSW, of the Chinese peanut mini-core collection were measured in four environments (E1–E4). All the traits exhibited a large phenotypic variation (Table 1 and Supplementary Figure 2). The trait of HSW showed the highest CV (average: 28.97%), while the trait of SW showed the lowest CV (average: 10.46%). Among environments, the highest CV (30.41%) was observed for HSW in E1 (range: 24–117 g; mean: 63 g), while the lowest CV (10.55%) was found for SW in E4 (range: 0.65–1.2 cm; mean: 0.92 cm) (Table 1).

**TABLE 1:** Phenotypic performance of the six yield-related traits in the Chinese peanut mini-core collection under four environments.

Trait	Environment	Minimum	Maximum	Mean	SD	CV (%)	*H*^2^
PL (cm)	E1	1.94	7.25	3.20	0.61	19.06	0.92
	E2	2.08	3.91	3.07	0.45	14.66	
	E3	2.16	4.47	3.34	0.52	15.57	
	E4	1.89	4.25	3.10	0.49	15.81	
PW (cm)	E1	1.20	1.98	1.42	0.17	11.97	0.94
	E2	1.01	1.89	1.43	0.17	11.89	
	E3	1.00	2.19	1.47	0.19	12.93	
	E4	1.04	2.21	1.43	0.18	12.59	
SL (cm)	E1	0.89	2.44	1.52	0.28	18.42	0.96
	E2	1.04	2.07	1.56	0.24	15.38	
	E3	1.08	2.21	1.66	0.24	14.46	
	E4	1.05	2.07	1.57	0.25	15.92	
SW (cm)	E1	0.61	1.20	0.91	0.12	13.19	0.91
	E2	0.63	1.01	0.84	0.07	8.33	
	E3	0.67	1.19	0.95	0.10	10.53	
	E4	0.65	1.20	0.92	0.09	9.78	
HPW (g)	E1	68.00	346.00	168.10	48.14	28.64	0.96
	E2	69.67	250.13	144.47	40.30	27.90	
	E3	51.10	364.25	187.59	50.80	27.08	
	E4	53.46	308.43	161.44	43.60	27.01	
HSW (g)	E1	24.00	117.00	63.00	19.16	30.41	0.94
	E2	26.19	107.27	56.40	16.38	29.04	
	E3	28.00	116.80	70.74	19.51	27.58	
	E4	23.27	110.98	59.36	17.12	28.84	

*PL, pod length; PW, pod width; SL, seed length; SW, seed width; HPW, hundred-pod weight; HSW, hundred-seed weight; E1, Nanchong in 2015; E2, Wuhan in 2015; E3, Nanchong in 2016; E4, Wuhan in 2016; SD, standard deviation; CV, coefficients of variation; *H*^2^, broad-sense heritability.*

The broad-sense heritability was 0.92, 0.94, 0.96, 0.91, 0.96, and 0.94 for PL, PW, SL, SW, HPW, and HSW, respectively (Table 1). The high heritability indicated that genetic factors played a predominant role in determining the variation for these traits. To pinpoint their relationships, correlation coefficients were calculated between the traits across environments (Table 2). Highly positive correlations were observed in all pairs of the six yield-related traits across environments (Table 2). The highest correlation coefficient was observed between HPW and HSW, which varied from 0.90 in E1 to 0.92 in E4, with an average of 0.91 (Table 2). The strong and stable relationship with each other across environments indicated that these traits might be affected by common genetic factors.

**TABLE 2:** Correlation analysis for the six traits across multi-environments.

Trait	Environment	PL	PW	SL	SW	HPW
PW	E1	0.67^∗∗^				
	E2	0.71^∗∗^				
	E3	0.71^∗∗^				
	E4	0.79^∗∗^				
SL	E1	0.66^∗∗^	0.70^∗∗^			
	E2	0.83^∗∗^	0.78^∗∗^			
	E3	0.82^∗∗^	0.78^∗∗^			
	E4	0.85^∗∗^	0.80^∗∗^			
SW	E1	0.45^∗∗^	0.70^∗∗^	0.73^∗∗^		
	E2	0.28^∗∗^	0.64^∗∗^	0.55^∗∗^		
	E3	0.51^∗∗^	0.83^∗∗^	0.72^∗∗^		
	E4	0.48^∗∗^	0.77^∗∗^	0.66^∗∗^		
HPW	E1	0.71^∗∗^	0.88^∗∗^	0.74^∗∗^	0.74^∗∗^	
	E2	0.82^∗∗^	0.87^∗∗^	0.83^∗∗^	0.63^∗∗^	
	E3	0.80^∗∗^	0.92^∗∗^	0.85^∗∗^	0.84^∗∗^	
	E4	0.83^∗∗^	0.91^∗∗^	0.85^∗∗^	0.83^∗∗^	
HSW	E1	0.62^∗∗^	0.81^∗∗^	0.82^∗∗^	0.86^∗∗^	0.90^∗∗^
	E2	0.70^∗∗^	0.85^∗∗^	0.87^∗∗^	0.74^∗∗^	0.91^∗∗^
	E3	0.68^∗∗^	0.87^∗∗^	0.87^∗∗^	0.91^∗∗^	0.91^∗∗^
	E4	0.68^∗∗^	0.85^∗∗^	0.86^∗∗^	0.93^∗∗^	0.92^∗∗^

PL, pod length; PW, pod width; SL, seed length; SW, seed width; HPW, hundred-pod weight; HSW, hundred-seed weight; E1, Nanchong in 2015; E2, Wuhan in 2015; E3, Nanchong in 2016; E4, Wuhan in 2016.

**P < 0.01.

### Genomic Variation

**FIGURE 1:**
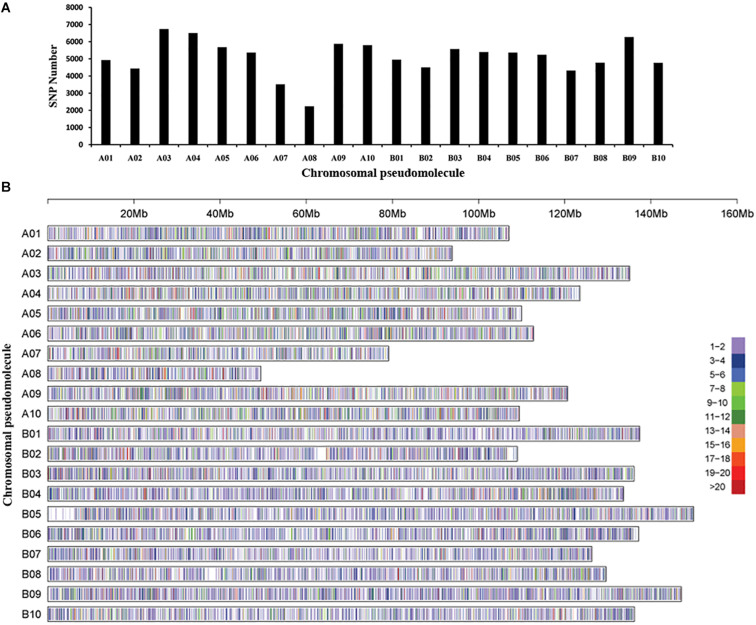
Single-nucleotide polymorphism (SNP) density and distribution across the genome of the peanut. **(A)** The distribution of the number of SNPs on each chromosomal pseudomolecule. **(B)** SNP density on each chromosomal pseudomolecule. The horizontal axis shows the length of the pseudomolecule; the number of SNPs per 20-kb window is shown as color index.

The GBS library was constructed for each accession, and massively parallel sequencing was performed to generate ∼311 Gb data and 589,167,078 tags. Polymorphic reads defined by GATK software and a total of 3,070,141 SNPs were initially identified among the 250 peanut accessions. After filtering, a total of 105,814 SNPs were retained. The chromosomal pseudomolecule A03 (6,737 SNPs) had the largest number of SNPs, followed by A04 (6,502 SNPs), while the smallest number of SNPs was detected on chromosomal pseudomolecule A08 (2,233 SNPs), followed by A07 (3,518 SNPs) (Figures 1A,B). Functional annotation detected ∼86% SNPs located in intergenic regions, while 14% SNPs were in genic regions. The largest number of SNPs in genic regions was in introns of annotated genes followed by untranslated regions and coding regions of annotated genes (Supplementary Table 3). A total of 2,085 SNPs were found in the coding regions, and 3,688 SNPs were in the gene upstream regions.

The LD was estimated using the *r*^2^ of SNP pairs. Decay of *r*^2^ value was analyzed across all chromosomes. Almost 25.7% *r*^2^ showed statistical significance (*P* < 0.05). The value where *r*^2^ dropped to half of its maximum was set as the background level. The extent of LD was found in the peanut panel at approximately 1.3 Mb, which is the point of 50% decay *r*^2^ value (*r*^2^ = 0.41; Supplementary Figure 3).

### Population Structure Analysis

**FIGURE 2:**
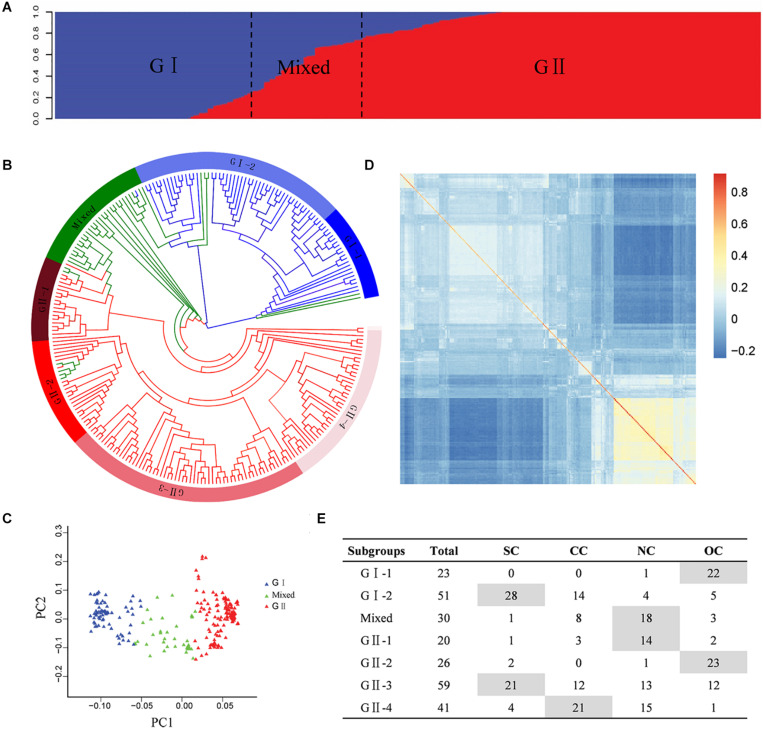
Genetic structures of the Chinese peanut mini-core collection. **(A)** Population structure. **(B)** Phylogenetic tree. The layer rings indicate the group and subgroup name. **(C)** Principal component analysis. **(D)** Heat map of pairwise relative kinship estimates. **(E)** The geographic origin of each accession in each cluster. SC, southern China; CC, central China; NC, northern China; OC, countries other than China. The information of botanical subspecies, origin geographic region, and group classification of each accession is provided in Supplementary Table 1.

The population structure analysis was estimated based on the SNP genotype of the Chinese peanut mini-core collection. Bayesian clustering analysis detected significant changes in both delta *k*-value and LnP(*D*) value when *k* was increased from 1 to 2, suggesting two as the most likely number of inferred clusters. The first group, called G I, comprised 74 accessions, of which 64 accessions (86.49%) were subspecies of ssp. *fastigiata*. The second group, named G II, included 146 accessions, of which 95 accessions (65.07%) were subspecies of spp. *hypogaea*. In the mixed group, there were 15 accessions (50%) from ssp. *fastigiata* and 13 accessions (43.33%) from ssp. *hypogaea* (Figure 2A, Supplementary Table 1, and Supplementary Figure 4). In spite of the discrepancies, the population structure was obviously associated with the classification of botanical subspecies (Supplementary Table 1 and Supplementary Figure 4). Based on color system classification, the comparison of the phylogenetic tree and structure analysis (Figure 2B) showed that the majority accessions of a group in structure analysis also showed close phylogenetic relationships in the neighbor-joining tree analysis. The PCA plot of the top two principal components of this population based on structure result also indicated a clear separation (Figure 2C). In the association panel, a kinship coefficient less than 0.25 accounted for 95.23%, indicating that most of the accessions had a weak relationship (Supplementary Figure 5). The *K* matrix was visualized using a heat map, in which the groups were clearly separated (Figure 2D).

According to phylogenetic relationships and stated location of origin, accessions in each group were further classified into several subgroups, which exhibited obviously geographic distribution patterns (Figures 2B,E and Supplementary Table 1). The G I group was classified into two subgroups, with G I-1 mainly containing ssp. *fastigiata* accessions from countries other than China and G I-2 mainly consisting of ssp. *fastigiata* accessions from southern China. The G II group was classified into four subgroups, with G II-1 to G II-4 mainly consisting of ssp. *hypogaea* accessions from northern China, countries other than China, southern China, and central China, respectively (Figures 2B,E). The mixed group had accessions mainly from north China.

### Genome-Wide Association Studies

**FIGURE 3:**
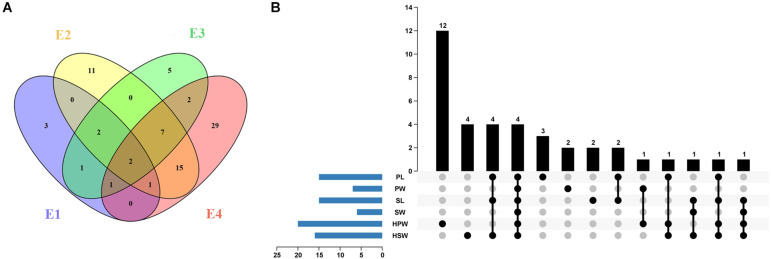
The profile of the associated loci for the six yield-related traits of the Chinese peanut mini-core collection under four different environments. **(A)** The Venn diagram of associated loci for six traits under four environments (E1–E4). **(B)** The statistical column diagram of the associations of the six yield-related traits and their interactive upset plot. E1, Nanchong in 2015; E2, Wuhan in 2015; E3, Nanchong in 2016; E4, Wuhan in 2016; PL, pod length; PW, pod width; SL, seed length; SW, seed width; HPW, hundred-pod weight; HSW, hundred-seed weight.

A total of 10, 38, 20, and 57 significantly associated SNP loci (*P* < 4.73 × 10^–7^) were identified for the six yield-related traits under four environments of E1, E2, E3, and E4, respectively (Figure 3A and Supplementary Table 4). The larger number of association loci of a single trait in each environment was 15 for SL in E4, 14 for PL in E4, and 14 for HSW in E1, while no significant association could be detected for SW in E2 and SL in E1 and E3 (Supplementary Table 4). For each trait, a total of 15, 7, 15, 6, 20, and 16 non-redundant associations were identified for PL, PW, SL, SW, HPW, and HSW, respectively (Figure 3B and Supplementary Tables 4, 5). Most of the associations were distributed on chromosomal pseudomolecules A02, A06, B04, and B06, while a small number of associations were detected on A03, A04, A05, B07, B08, B09, and B10. The Manhattan plots and quantile–quantile plots of each trait under different environments are shown in Supplementary Figures 6–11.

Among the 79 significantly associated loci identified for the six yield-related traits (Figure 3A and Supplementary Table 5), 31 were detected in at least two environments (Figure 3A and Supplementary Table 6). The associated marker A02-86439145 for PW and HSW was repeatedly detected across four environments, while 11 other such associated markers, such as B06-129049126 for PL and B04-14788349 for PW and HSW, were detected across three environments. A set of 15 markers was found to be associated with multiple traits (Figure 3B and Supplementary Table 7). Of these, four markers (A02-86439145, A06-108577126, A06-108613799, and B04-14788349) were found to be significantly associated with all six yield-related traits. Six markers (A06-108797911, B06-127760084, B06-128026082, B06-128423460, B06-129334953, and B06-129337040) were significantly associated with three of these traits. The high and positive phenotypic correlations among the yield-related traits and the common associated markers detected by GWAS suggested that it was possible to improve the multiple traits of seed (pod) size and weight through GAB.

### Stable Major Candidate Genomic Regions

The markers A06-108577126 and A02- 86439145 were repeatedly detected to be significantly associated with all six yield-related traits. The marker A06-108577126 was identified with ∼20% phenotypic variation explained (PVE) of PW in three environments and of HPW, HSW, PL, SL, and SW in two environments (Table 3). The highest phenotypic variance explained by this significant association was 36.27% for SL in E2. A similar phenomenon was observed with the marker A02-86439145 (Table 3). The results suggested that the candidate genomic regions on A06 and A02 might contain major and stable loci regulating the yield-related traits.

**TABLE 3:** The repeated detected major association loci (*P* < 4.73 × 10^–7^).

SNP	Reference	Tother	Trait	Environment	Maf	*P*-value	PVE (%)
A06-108577126	C	T	PL	E2	0.49	8.73	16.96
				E3	0.49	6.58	24.27
			PW	E2	0.49	10.07	19.33
				E3	0.49	6.50	15.90
				E4	0.49	9.31	20.12
			SL	E2	0.49	10.12	36.27
				E4	0.49	11.34	23.12
			SW	E3	0.49	6.38	16.79
				E4	0.49	8.23	20.28
			HPW	E2	0.49	10.21	19.40
				E3	0.49	7.37	17.69
			HSW	E2	0.49	12.16	25.96
				E3	0.49	6.98	21.19
A02- 86439145	G	T	PL	E2	0.19	7.92	17.83
				E4	0.19	8.65	22.43
			PW	E2	0.19	8.49	24.76
				E3	0.19	6.96	21.59
				E4	0.19	6.56	20.70
			SL	E4	0.19	7.48	23.66
			SW	E3	0.19	6.49	19.20
				E4	0.19	6.54	19.66
			HPW	E2	0.19	8.73	29.55
				E3	0.19	7.56	23.43
			HSW	E2	0.19	8.28	26.62
				E3	0.19	7.04	19.77

*E1, Nanchong in 2015; E2, Wuhan in 2015; E3, Nanchong in 2016; E4, Wuhan in 2016; PL, pod length; PW, pod width; SL, seed length; SW, seed width; HPW, hundred-pod weight; HSW, hundred-seed weight; PVE, phenotypic variation explained by each significant marker. *P*-value represents −log_10_(*P*).*

Candidate genes were selected in the genomic regions if they encode components of metabolic or signaling pathways known to be related to the corresponding phenotypes or based on the expression profile (for example, tissue-specific expression) of peanut (Clevenger et al., 2016). A total of 17 and 29 candidate genes for the yield traits were discovered in the genomic regions underlying the A06-108577126 and A02-86439145, respectively (Table 4). The *AP2* transcription factor (*Aradu.GG3LG*) is the homologous gene of *AINTEGUMENTA* (*ANT*, *AT4G37750*) which has been reported to regulate seed size through regulating the growth of the embryo organ and the proliferation of cell number in *Arabidopsis* (Mizukami and Fischer, 2000). The EamA-like transporter family protein (*Aradu.K0L5G*) was found to be specifically expressed in seeds and pods according to expression profile using RNA sequencing data from 22 tissues of *A. hypogaea* L. (Clevenger et al., 2016). A set of 16 candidate genes was predicted to code for cytochrome P450s, which were reported to be involved in the regulation of cell proliferation in the embryo and endosperm (Zhang et al., 2012; Nagasawa et al., 2013). A set of 13 candidate genes was predicted to code for receptor kinases, which were reportedly involved in the *CLAVATA–WUSCHEL* (*CLV-WUS*) pathway that affected seed size by controlling the locule number (Somssich et al., 2016). Seven genes were predicted to code for transcription factors, which controlled fruit size by affecting the fertility, seed number, cell number, and cell size (De Folter et al., 2004; Swaminathan et al., 2008; Zhang et al., 2016). Two candidate genes were predicted to code for ubiquitin protein, which were reported to regulate fruit size by influencing cell proliferation (Wang Z. et al., 2016). Two genes were predicted to code for beta-fructofuranosidases, which were reported to affect seed size by affecting cell division and cell elongation (Jofuku et al., 2005). A single gene each encoded G-protein, cytokinin dehydrogenase, sugar transport protein, and UDP-glycosyltransferase protein, which was reported in regulating seed size (Hussain et al., 2020).

**TABLE 4:** The putative candidate genes in the two stable and major candidate genomic regions.

Traits	Associated SNP loci (*P* < 4.73 × 10^–7^)	Position in diploid genomes		Gene model	Position of genes in tetraploid genome (AABB)			Functional annotation
					
		Chr.	Candidate genomic region		Chr.	Start	End	
PL, PW, SL, SW, HPW, HSW	A06-108577126	A06	107277126-109877126	Aradu.VE705	Arahy.06	112677427	112678982	WRKY transcription factor
				Aradu.KD2FK	Arahy.06	112709403	112712304	WRKY transcription factor
				Aradu.9J6N2	Arahy.06	113104568	113107684	Ubiquitin hydrolase
				Aradu.7ZU9R	Arahy.06	113174524	113178801	Ubiquitin hydrolases
				Aradu.K0L5G	Arahy.06	113197631	113202441	EamA-like transporter
				Aradu.1P9QN	Arahy.06	113332596	113338761	Ubiquitin hydrolase
				Aradu.Y12B3	Arahy.06	113536062	113541907	G-protein coupled receptor
				Aradu.T9MSY	Arahy.06	113648657	113650961	WRKY transcription factor
				Aradu.D11TE	Arahy.06	113843579	113850297	Cytokinin dehydrogenase
				Aradu.J439W	Arahy.06	114081915	114090053	Cytochrome P450
				Aradu.ET296	Arahy.06	114159451	114163839	Beta-fructofuranosidase
				Aradu.T5FHF	Arahy.06	114175030	114179576	Beta-fructofuranosidase
				Aradu.XGK7G	Arahy.06	114305671	114310826	Receptor kinase 5
				Aradu.HD3ZK	Arahy.06	99108009	99109852	Sugar transport protein
				Aradu.MN4MZ	Arahy.06	115297441	115302375	Transcription factor bHLH74
				Aradu.GG3LG	Arahy.06	115340494	115345619	AP2 transcription factor ANT
				Aradu.75PY6	Arahy.06	115413670	115419119	Growth-regulating factor
PL, PW, SL, SW, HPW, HSW	A02-86439145	A02	85139145-87739145	Aradu.398F4	Arahy.02	94217863	94224568	UDP-glycosyltransferase protein
				Aradu.1WD61	Arahy.02	94533579	94536262	Cytochrome P450
				Aradu.6V2WY	Arahy.02	94572810	94576010	Cytochrome P450
				Aradu.55VHH	Arahy.02	94572810	94576010	Cytochrome P450
				Aradu.TNI2G	Arahy.02	94589384	94591342	Cytochrome P450
				Aradu.HP9JD	Arahy.02	94597039	94599985	Cytochrome P450
				Aradu.32QV2	Arahy.02	94611083	94613464	Cytochrome P450
				Aradu.RP8T4	Arahy.02	94620947	94623505	Cytochrome P450
				Aradu.KQ6NP	Arahy.02	94704533	94708850	Cytochrome P450
				Aradu.LM4BA	Arahy.02	94672422	94676561	Cytochrome P450
				Aradu.YXE38	Arahy.02	94704533	94708850	Cytochrome P450
				Aradu.TG31C	Arahy.02	95164227	95183544	Receptor-like protein kinase
				Aradu.5VY9W	Arahy.02	95164227	95183544	Receptor-like protein kinase
				Aradu.CI98G	Arahy.02	95268925	95272603	Receptor-like protein kinase
				Aradu.V72UR	Arahy.04	5155978	5158411	Receptor-like protein kinase
				Aradu.UH1VC	Arahy.02	95318115	95319019	Receptor-like protein kinase
				Aradu.F42D3	Arahy.02	95348737	95351160	Receptor-like protein kinase
				Aradu.D2PD6	Arahy.02	95354093	95356282	Receptor-like protein kinase
				Aradu.AT4A3	Arahy.02	95433341	95435592	Receptor-like protein kinase
				Aradu.L44LL	Arahy.02	95354093	95356282	Receptor-like protein kinase
				Aradu.T5WNW	Arahy.02	95433341	95435592	Receptor-like protein kinase
				Aradu.T3V5H	Arahy.02	95436259	95438492	Receptor-like protein kinase
				Aradu.55F3D	Arahy.02	95456281	95459774	Receptor-like protein kinase
				Aradu.04TM0	Arahy.02	95596105	95599322	Cytochrome P450
				Aradu.300HC	Arahy.02	96425710	96429083	Growth-regulating factor
				Aradu.2I26G	Arahy.02	96476727	96482536	Cytochrome P450
				Aradu.SLR60	Arahy.02	96476727	96482536	Cytochrome P450
				Aradu.BI3QN	Arahy.02	96499325	96503464	Cytochrome P450
				Aradu.9JB4Z	Arahy.02	96558840	96562030	Cytochrome P450

*Chr., chromosome.; PL, pod length; PW, pod width; SL, seed length; SW, seed width; HPW, hundred-pod weight; HSW, hundred-seed weight.*

### Diagnostic Marker

**FIGURE 4:**
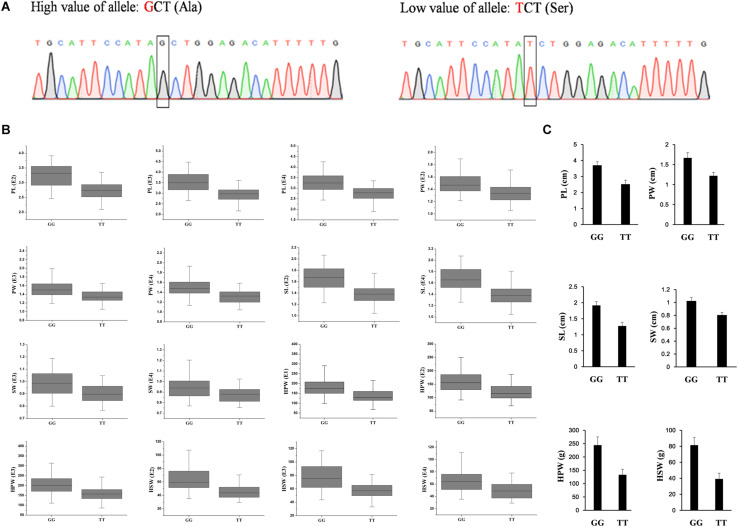
The validated diagnostic marker on A06 for the six yield-related traits. **(A)** The partial sequence diagrams included the diagnostic locus of the non-synonymous single-nucleotide polymorphism (G to T) after amplifying by polymerase chain reaction and sequencing. **(B)** The allelic effect at A06-107901527 for the significant associated target trait in different environments (*P* < 0.001) in the association panel. **(C)** The accessions with GG alleles show a significantly higher value of seed (pod) size and weight than those with TT allele (*P* < 0.001, Student’s *t*-test) in two extreme trait groups of peanuts. PL, pod length; PW, pod width; SL, seed length; SW, seed width; HPW, hundred-pod weight; HSW, hundred-seed weight; E1, Nanchong in 2015; E2, Wuhan in 2015; E3, Nanchong in 2016; E4, Wuhan in 2016.

To validate the diagnostic marker for seed (pod) size and weight, we investigated the correlation between nucleotide polymorphism in the candidate genes and phenotypic variation in the association panel. There were 15 non-synonymous SNPs in seven candidate genes in the genomic region underlying the A06-108577126 and 23 non-synonymous SNPs in eight candidate genes in the genomic region underlying the A02-86439145. The allelic effect of each genotypic class for the associated target trait was estimated in different environments. Unfortunately, we did not find non-synonymous SNP to correlate with phenotypic variation in the genomic region underlying the A02-86439145, but in the genomic region underlying the A06-108577126, we found the non-synonymous SNP (G→T, Ala→Ser, located in *Aradu.K0L5G*) at A06-107901527 (Figure 4A) which showed that the GG genotype contained a significantly higher (*P* < 0.001) seed (pod)-related trait value than those having TT genotype in our panel under different environments (Figure 4B).

In addition, two diverse extreme trait groups of 25 accessions with high trait values and 25 accessions with low trait values of seed (pod) size and weight were surveyed using SSR primers that amplified a fragment including the SNP. PCR product sequencing revealed a clear differentiation between the accessions with high and low values of seed (pod)-related traits, with the high value accessions having the GG genotype and the low value accessions having the TT genotype (Figure 4C). The results indicated that the SNP of Aradu*-*A06-107901527 could be used as a diagnostic marker for seed (pod) size and weight in peanut and would be useful for marker-assisted breeding of high-yield peanut varieties.

## Discussion

The Chinese peanut mini-core collection was selected from 6,390 peanut germplasm resources in our previous study (Jiang et al., 2008). Here we used an abundant diversity of the collection (Jiang et al., 2008, 2010, 2014) combined with GBS sequencing for genetic mapping to dissect the genetic basis of yield traits. The method has been shown to provide sufficient power to map major effect loci, even to the level of individual causal SNPs in some previous studies (Wang H. et al., 2016). Fortunately, the high-quality reference genomes for both peanut progenitors, *A. duranensis* (A genome) and *A. ipaensis* (B genome) (Bertioli et al., 2016; Chen X. et al., 2016; Lu et al., 2018), are available with annotations. Assemblies of the reference genome have also become available for cultivated peanut (Bertioli et al., 2019; Chen et al., 2019; Zhuang et al., 2019) and will increase the efficiency of such studies in the future. In this study, we have used progenitor genome sequence assemblies for calling SNPs in the Chinese peanut mini-core collection as reported in some recent publications (Chavarro et al., 2019; Gangurde et al., 2020; Huang et al., 2020; Yu et al., 2020). Finally, 105,814 high-quality genome-wide SNPs were retained in this study, which is, so far, the highest number of markers used for performing association analysis in peanut.

Population structure is an important component in association mapping analysis, and it helps in reducing the detection of false positives among associated markers. The structure analysis classified the Chinese peanut mini-core collection into two groups and a mixed one. The peanut germplasm collections in previous studies could be divided into two to four groups, which were always associated with different botanical subspecies or botanical types (Wang et al., 2011; Jiang et al., 2014; Zhang et al., 2017; Zheng et al., 2018). In the present study, most of the accessions of two subspecies of ssp. *fastigiata* and ssp. *hypogaea* could be clearly divided into GI and GII, respectively, but it was hard to clearly distinguish the botanical types because many accessions in this population harbored features of mixed morphological types. The phenomenon may be due to the genetic exchange between varieties from different botanical types. Further analysis found that G I contained two subgroups of ssp. *fastigiata* accessions mainly from southern China and countries other than China, while G II contained four subgroups of ssp. *hypogaea* accessions mainly from northern China, southern China, central China, and countries other than China, suggesting that obvious geographical distribution patterns existed in the subgroups. A similar phenomenon was observed in many other crops, such as rice, soybean, and sorghum (Morris et al., 2013; Zhou et al., 2015; Wang H. et al., 2019). The result suggested that different geographic origins of accessions harbored different genetic characteristics, probably enabling peanut accessions to adapt to various ecological environments.

In this study, 79 significantly associated loci were identified for six yield-related traits (Figure 3B and Supplementary Table 5). The number of QTLs detected for seed (pod) size and weight was significantly higher than that identified in previous genetic mapping studies (Huang et al., 2015; Zhang et al., 2017; Zhao et al., 2017). Some of the associated loci identified in this study were consistent with QTLs obtained from segregating populations. The marker A02-86439145 was found to be associated with all six yield-related traits. The SNP A02-86439292 was another associated marker located 147 bp away from SNP A02-86439145 and explained 20.85% of the phenotypic variance for PL in E4. The genomic region underlying A02-86439145 and A02-86439292 was located within the QTL of *q100SW2* for HSW in one environment (PVE: 24.69%) and *qSL2* for SL in four environments (PVE ranged from 42.43 to 61.74%) in Huayu36 × 6-13 population (Zhang et al., 2019). Another significant associated marker B04-14788349 was detected to be associated with SW and HPW in two environments, PW and HSW in three environments, and PL and SL in a single environment. The genomic region underlying B04-14788349 was co-localized within the *qSLB04* for SL, *qSWB04.3* for SW, and *qHSWB04.4* for HSW of the Zhonghua 5 × ICGV 86699 population (Zhou, 2018). Moreover, 18 associations were consistently detected in two environments, 11 associations were repeatedly detected in three environments, and two associations were detected across four environments (Figure 3A and Supplementary Table 6). The associations detected in different populations or environments achieved mutual corroboration, indicating that these associated loci were stable and reliable. On the other hand, most of the associated loci identified in this study were new, such as A06-108577126 for the six yield-related traits and B06-127760084 for HSW, PL, and SL. The results showed that GBS-based GWAS was effective in revealing the genetic basis of seed (pod) size and weight.

Two important stable major associations were identified on pseudomolecules A06 and A02, and the PVE of the associations for yield-related traits could reach about 20% under multi-environments (Table 3). Our analysis also suggested 1.3 Mb LD distance in the association panel, which was similar to the results of two previous studies by Zhao et al. (2017) and Liu et al. (2020). In the candidate genomic region underlying A06-108577126, the *AP2* transcription factor (*Aradu.GG3LG*) was identified. Its homologous gene (*ANT*) had been well studied in *Arabidopsis*, and it controlled seed size through regulating the growth of the embryonic organ and the proliferation of cell number. However, information on the functional role of this gene in peanut is limited, making seed (pod) development a good concern in future research. In the same genomic region, the gene *Aradu.K0L5G* was found to be specifically expressed in seeds and pods. It was worth noting that the sequence polymorphism of the non-synonymous SNP located at A06-107901527 in *Aradu.K0L5G* was highly correlated with the phenotypic variation of the six yield-related traits. The SNP changed the base from G to T and resulted in the amino acid from Ala to Ser in *Aradu.K0L5G*. The homologous gene of *Aradu.K0L5G* in *Arabidopsis* (*At4g32140*) was reported to be a cell cycle-regulated gene, showing a significant fluctuation and a peak expression in the S phase (Menges et al., 2002). The two candidate genes represented excellently preferred candidates for follow-up analysis of causal polymorphisms and were ultimately confirmed by transformation. In the genomic regions underlying A06-108577126 and A02-86439145, no correlation between sequence variation and phenotypic variation was observed in the other 44 candidate genes for seed size and weight. Because the proteins encoded by these genes were reported to be involved in related pathways, these genes might be involved in regulating seed (pod) size and weight as well. We could not detect correlations between sequence variations and phenotypic variations in these genes, possibly because there were still not enough SNPs. Alternatively, some unknown genes or genomic components involved in trans-regulatory or epistatic interactions may be responsible for the indicated associated loci.

One of the main advantages of sequencing-based trait mapping approaches is the development of markers for target traits (Pandey et al., 2017). Diagnostic markers for some traits have been identified in peanut, such as rust resistance, late leaf spot resistance, and bacterial wilt resistance (Pandey et al., 2017; Luo et al., 2019). The diagnostic marker Aradu*-*A06-107901527 for seed (pod) size and weight was successfully validated in this study. Its sequence variation was correlated with phenotype variation and could be used to distinguish peanut accessions with corresponding high or low trait values. The allelic effect of the SNP was stable in multi-environments. This character allows breeders to avoid many difficulties in checking its stable performance in different places for higher field suitability. The diagnostic marker developed in this study could be applied to accelerate the marker-assisted breeding program of high-yield peanut varieties.

## Conclusion

In conclusion, the results provided a large data set of genomic variation for diverse varieties and a comprehensive presentation of associated loci for six yield-related traits in peanut. Putative candidate genes underlying stable major associated loci were discovered, and the correlation was investigated between nucleotide polymorphism and phenotype variation. The diagnostic marker was successfully validated and could be deployed in GAB of high-yield peanut varieties.

## Data Availability Statement

The datasets presented in this study can be found in online repositories. The names of the repositories and accession numbers can be found below: PRJNA687812 and PRJNA695811.

## Author Contributions

XZ and HJ conceived and designed the study. XZ and JG analyzed the data. XZ, LH, HL, NL, WC, YL, and BL performed the field trials and phenotyping. XZ wrote the manuscript. MP and RV revised and improved the manuscript. All authors read and approved the final manuscript.

## Conflict of Interest

The authors declare that the research was conducted in the absence of any commercial or financial relationships that could be construed as a potential conflict of interest.

## Footnotes

1 http://treesoft.sourceforge.net/treebest.shtml

2 http://cnsgenomics.com/software/gcta/

3 http://www.xzlab.org/software.html

4 https://www.peanutbase.org

5 https://primer3-sourceforge-net.caas.cn/

## Supplementary Material

Supplementary Figure 1 The geographic distribution of the accessions used in this study. Each accession is displayed as a dot on the world map. The composition (top right) of botanical subspecies and the geographic origin composition (bottom right) of the material panel used are shown.

Supplementary Figure 2 Frequency distribution of the phenotypic variation of the six traits in the Chinese peanut mini-core collection. The averaged phenotype value of four environments for each trait was used.

Supplementary Figure 3 Linkage disequilibrium decay for the Chinese peanut mini-core collection.

Supplementary Figure 4 The number of different botanical subspecies in each group.

Supplementary Figure 5 Distribution of pairwise relative kinship estimates. The values of kinship less than 0 were ordered as 0.

Supplementary Figure 6 Genome-wide association studies for pod length in four environments.

Supplementary Figure 7 Genome-wide association studies for pod width in four environments.

Supplementary Figure 8 Genome-wide association studies for seed length in four environments.

Supplementary Figure 9 Genome-wide association studies for seed width in four environments.

Supplementary Figure 10 Genome-wide association studies for hundred-pod weight in four environments.

Supplementary Figure 11 Genome-wide association studies for hundred-seed weight in four environments.

Supplementary Table 1 Summary of the Chinese peanut mini-core collection.

Supplementary Table 2 Specificities and missing data proportions of the genotype dataset before and after the missing genotypes are inferred.

Supplementary Table 3 Detailed list of the predicted effects of annotated single-nucleotide polymorphisms.

Supplementary Table 4 For each of the six yield-related traits, the number of single-nucleotide polymorphisms (SNPs) and the number of repeatedly detected SNPs in different environments.

Supplementary Table 5 Detailed information of the genome-wide significant association signals for the yield-related traits using the MLM (PCA + K) model.

Supplementary Table 6 The repeated detected single-nucleotide polymorphism for each trait in different environments.

Supplementary Table 7 The single-nucleotide polymorphism detected to be associated with multiple traits.
