# Supplementary material for: Dissection of the Genetic Basis of Yield-Related Traits in the Chinese Peanut Mini-Core Collection Through Genome-Wide Association Studies
Source: Front Plant Sci. 2021 May 20;12:637284. doi: 10.3389/fpls.2021.637284 (PMC8174301; doi:10.3389/fpls.2021.637284)
Supplement: Supplementary Figure 1 — The geographic distribution of the accessions used in this study. Each accession is displayed as a dot on the world map. The composition (top right) of botanical subspecies and the geographic origin composition (bottom right) of the material panel used are shown. [file Data_Sheet_1.PDF]

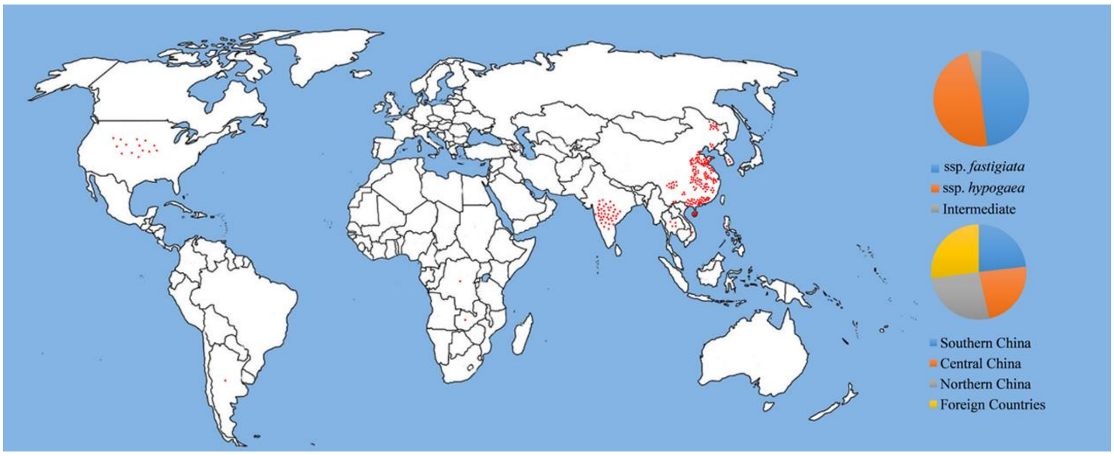

Supplemental Figure S1. The geographic distribution of the accessions used in this study. Each accession is displayed as a dot on the world map. The composition (top right) of botanical subspecies and the geographic origin composition (bottom right) of the used material panel were shown.

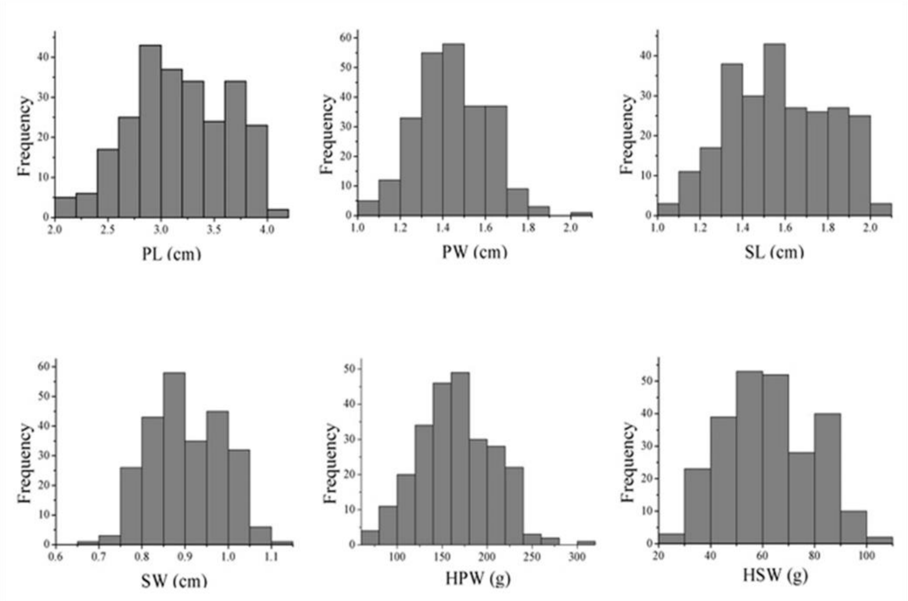

Supplemental Figure S2. Frequency distribution of phenotypic variation of the six traits in the Chinese peanut mini-core collection. The averaged phenotype value of four environments for each trait was used.

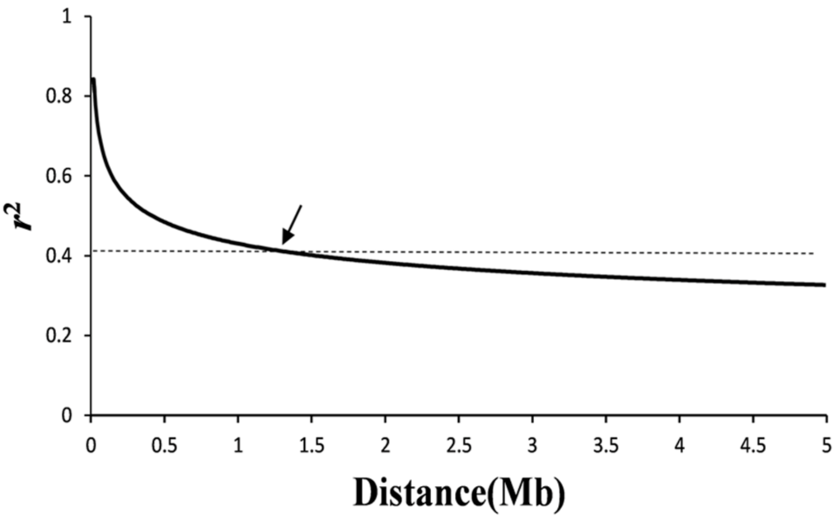

Supplemental Figure S3. Linkage disequilibrium decay for the Chinese peanut mini-core collection.

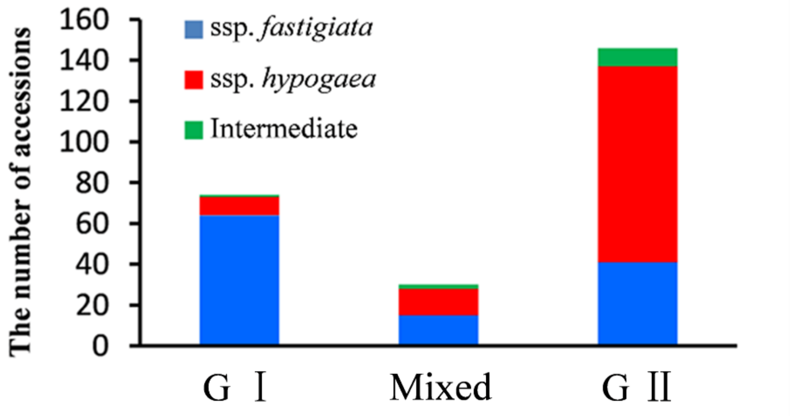

Supplemental Figure S4. The number of different botanical subspecies in each group.

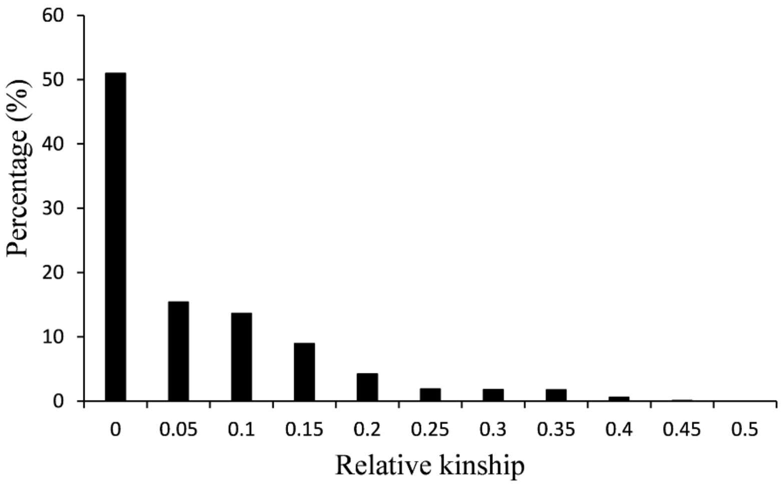

Supplemental Figure S5. Distribution of pairwise relative kinship estimates. The value of kinship less than 0 were ordered as 0.

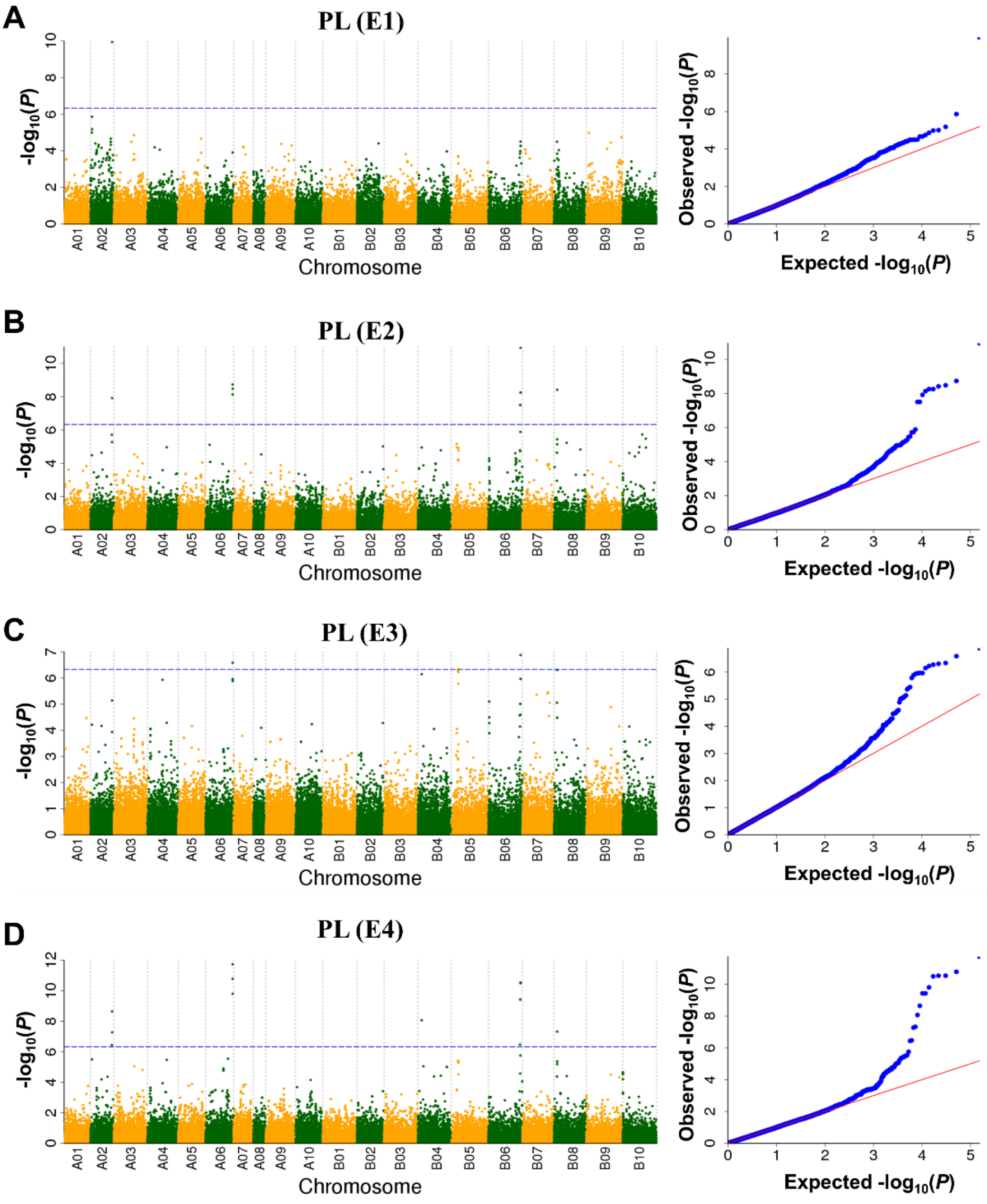

Supplemental Figure S6. Genome-wide association studies for PL in four environments. The significance threshold of $-\log_{10}(P)$ value is noted with the horizontal line. (A) Manhattan plot and quantile-quantile plot for PL in E1. (B) Manhattan plot and quantile-quantile plot for PL in E2. (C) Manhattan plot and quantile-quantile plot for PL in E3. (D) Manhattan plot and quantile-quantile plot for PL in E4. PL: pod length. E1: Nanchong in 2015; E2: Wuhan in 2015; E3: Nanchong in 2016; E4: Wuhan in 2016.

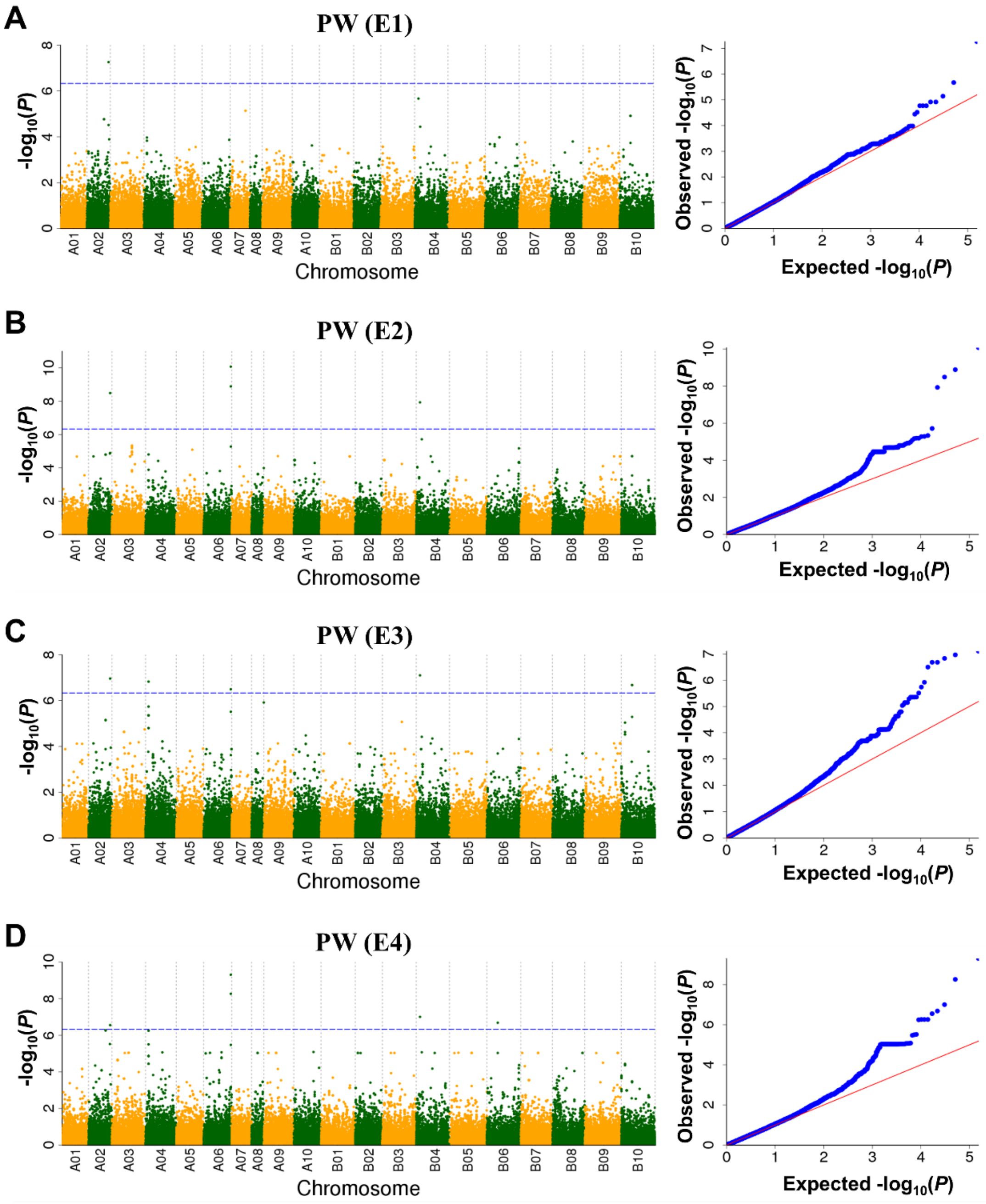

Supplemental Figure S7. Genome-wide association studies for PW in four environments. The significance threshold of $-\log_{10}(P)$ value is noted with the horizontal line. (A) Manhattan plot and quantile-quantile plot for PW in E1. (B) Manhattan plot and quantile-quantile plot for PW in E2. (C) Manhattan plot and quantile-quantile plot for PW in E3. (D) Manhattan plot and quantile-quantile plot for PW in E4. PW: pod width. E1: Nanchong in 2015; E2: Wuhan in 2015; E3: Nanchong in 2016; E4: Wuhan in 2016.

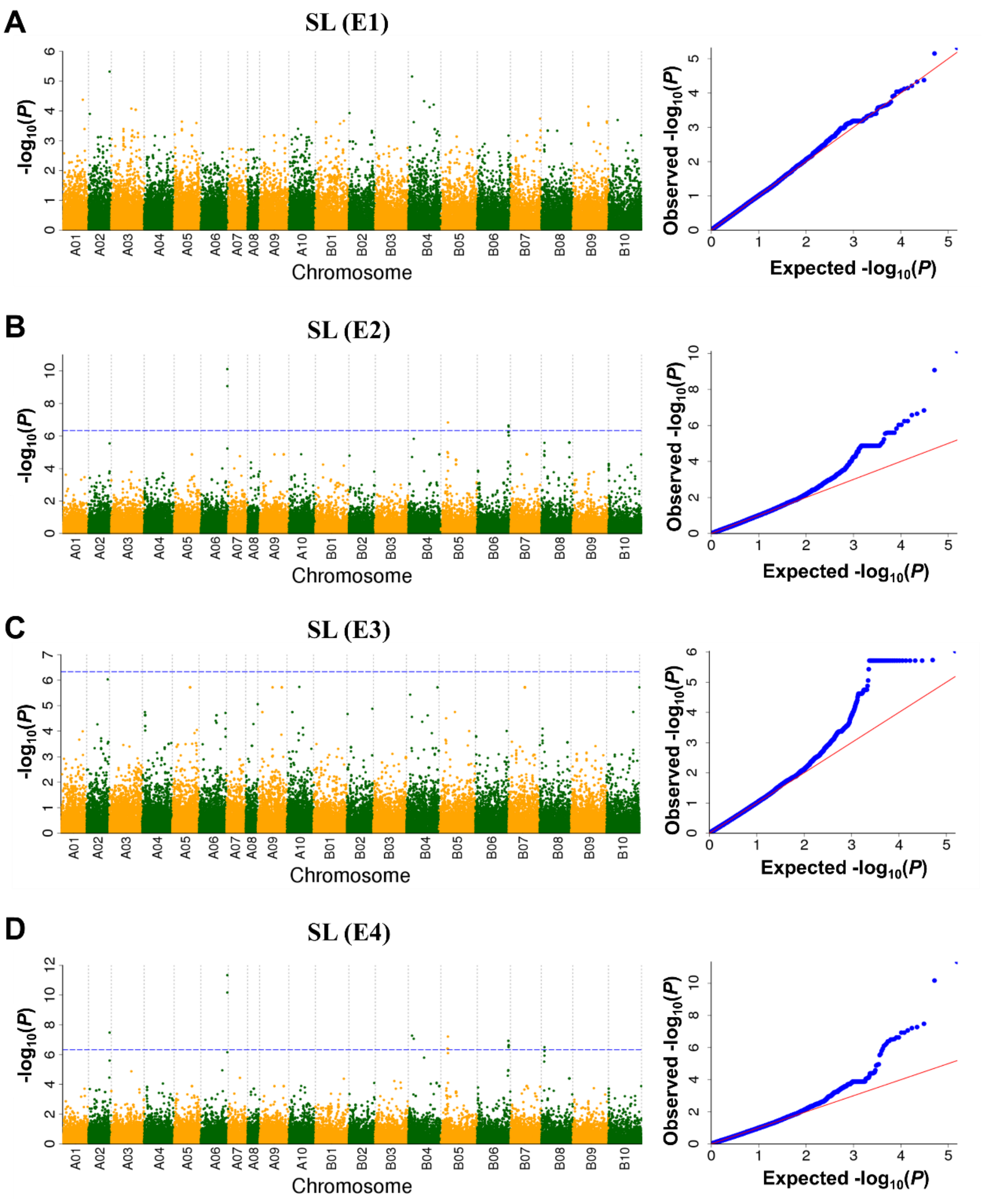

Supplemental Figure S8. Genome-wide association studies for SL in four environments. The significance threshold of –log$_{10}$(P) value is noted with the horizontal line. (A) Manhattan plot and quantile-quantile plot for SL in E1. (B) Manhattan plot and quantile-quantile plot for SL in E2. (C) Manhattan plot and quantile-quantile plot for SL in E3. (D) Manhattan plot and quantile-quantile plot for SL in E4. SL: seed length. E1: Nanchong in 2015; E2: Wuhan in 2015; E3: Nanchong in 2016; E4: Wuhan in 2016.

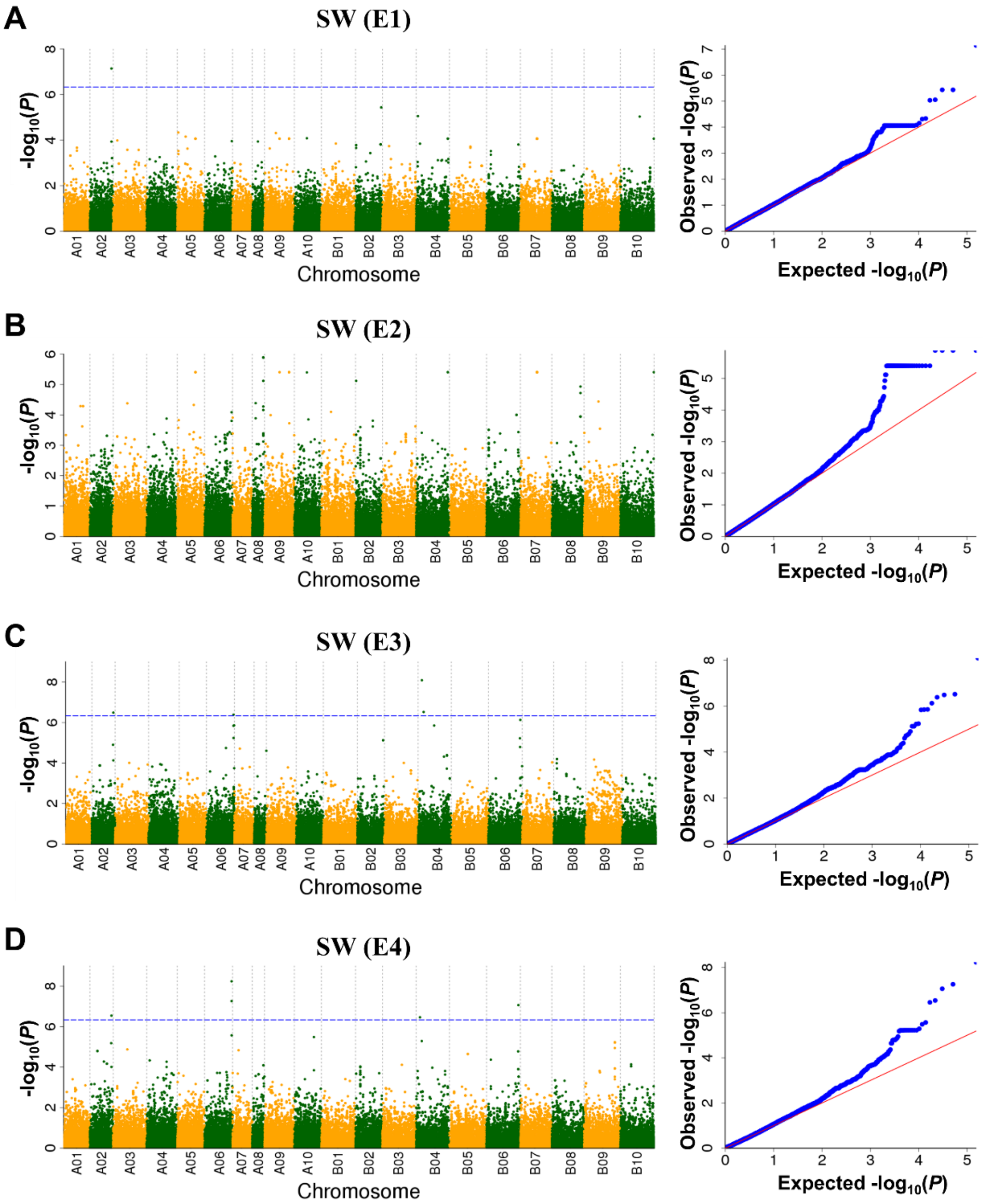

Supplemental Figure S9. Genome-wide association studies for SW in four environments. The significance threshold of $-\log_{10}(P)$ value is noted with the horizontal line. (A) Manhattan plot and quantile-quantile plot for SW in E1. (B) Manhattan plot and quantile-quantile plot for SW in E2. (C) Manhattan plot and quantile-quantile plot for SW in E3. (D) Manhattan plot and quantile-quantile plot for SW in E4. SW: seed width. E1: Nanchong in 2015; E2: Wuhan in 2015; E3: Nanchong in 2016; E4: Wuhan in 2016.

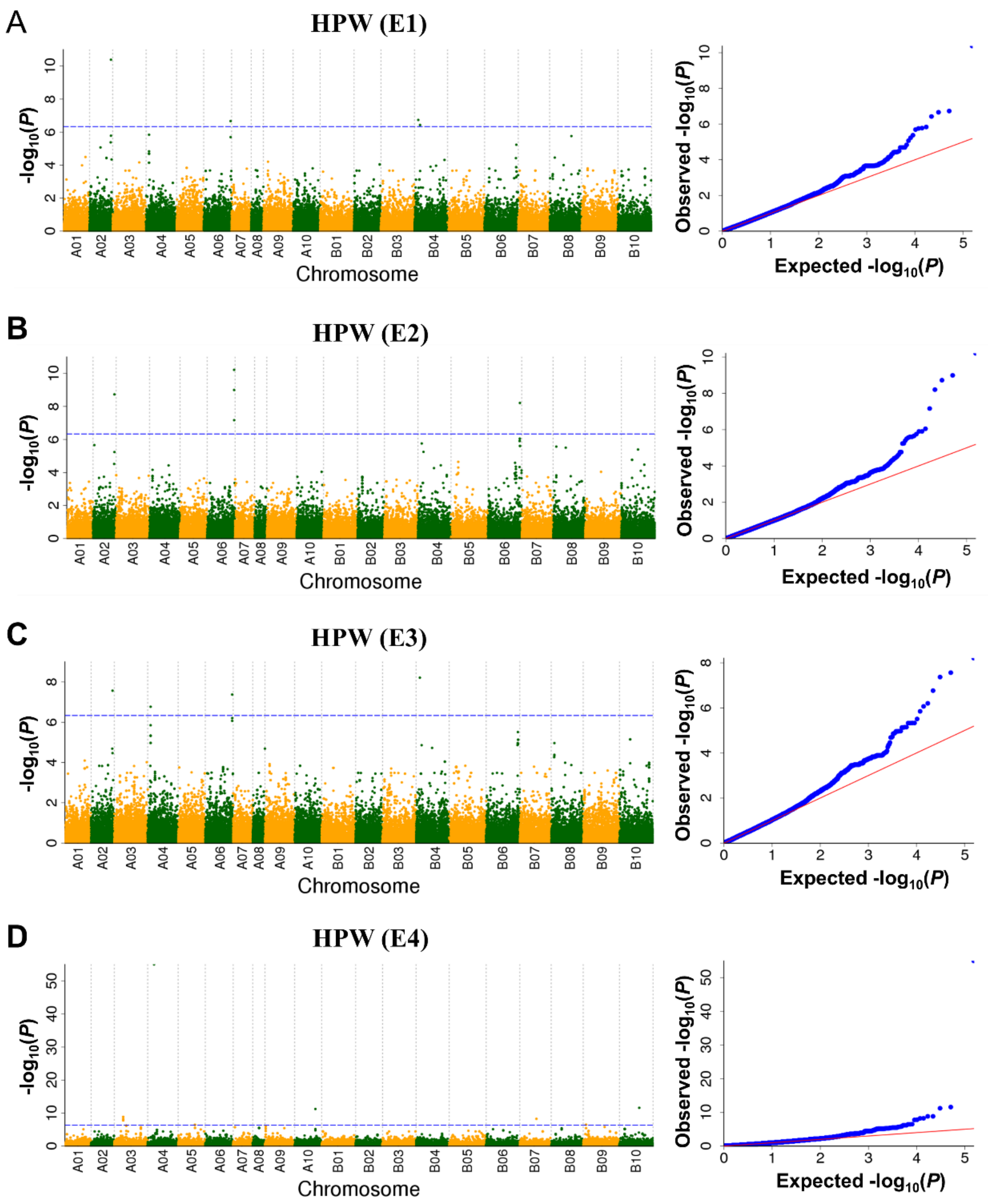

Supplemental Figure S10. Genome-wide association studies for HPW in four environments. The significance threshold of $-\log_{10}(P)$ value is noted with the horizontal line. (A) Manhattan plot and quantile-quantile plot for HPW in E1. (B) Manhattan plot and quantile-quantile plot for HPW in E2. (C) Manhattan plot and quantile-quantile plot for HPW in E3. (D) Manhattan plot and quantile-quantile plot for HPW in E4. HPW: hundred-pod weight. E1: Nanchong in 2015; E2: Wuhan in 2015; E3: Nanchong in 2016; E4: Wuhan in 2016.

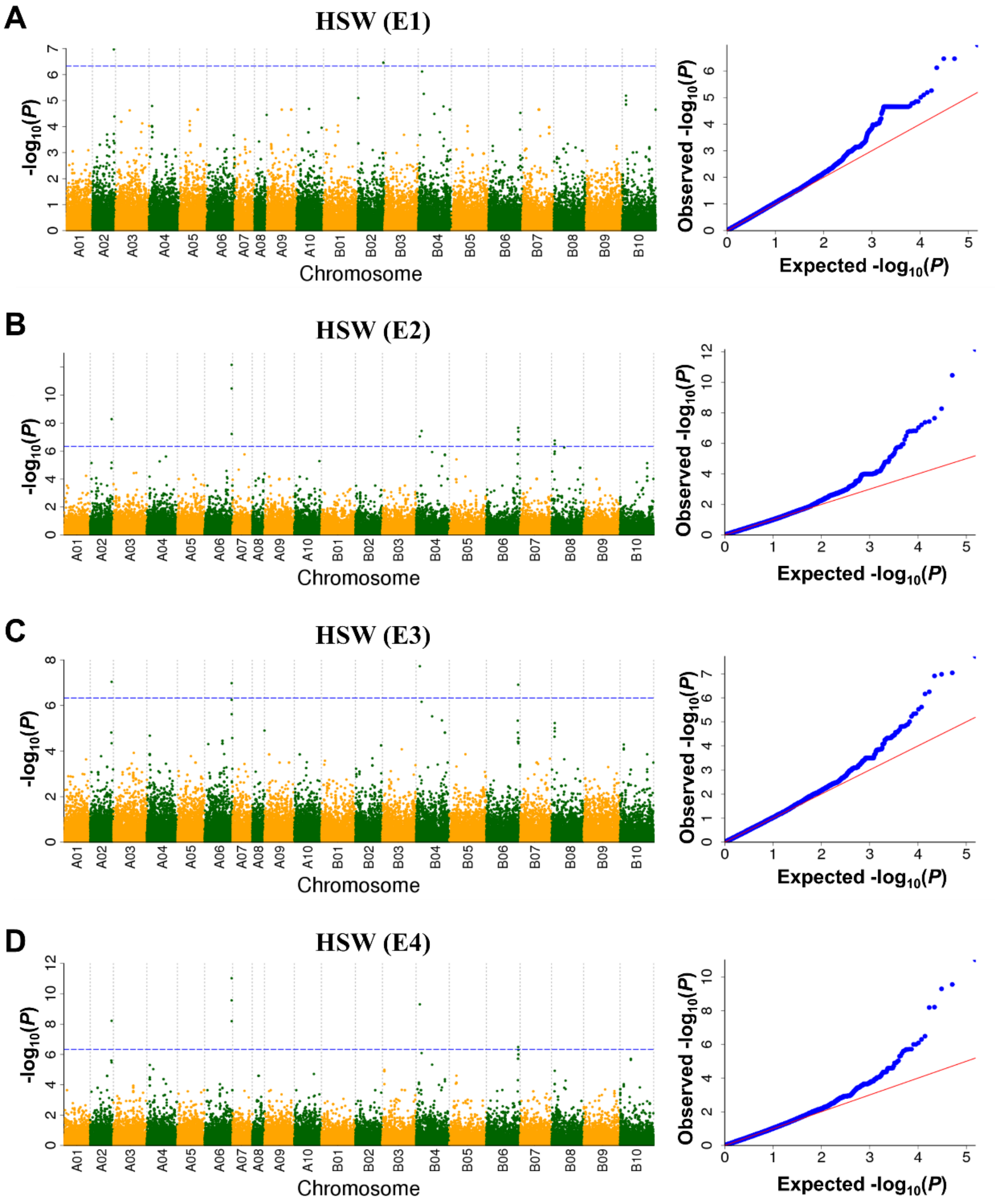

Supplemental Figure S11. Genome-wide association studies for HSW in four environments. The significance threshold of $-\log_{10}(P)$ value is noted with the horizontal line. (A) Manhattan plot and quantile-quantile plot for HSW in E1. (B) Manhattan plot and quantile-quantile plot for HSW in E2. (C) Manhattan plot and quantile-quantile plot for HSW in E3. (D) Manhattan plot and quantile-quantile plot for HSW in E4. HSW: hundred-seed weight. E1: Nanchong in 2015; E2: Wuhan in 2015; E3: Nanchong in 2016; E4: Wuhan in 2016.
